# Research progress on 40 Hz sensory stimulation for the treatment of Alzheimer’s disease

**DOI:** 10.3389/fnagi.2025.1710041

**Published:** 2026-01-16

**Authors:** Bing Tang, Ming Tao

**Affiliations:** 1 Second Clinical Medical School, Zhejiang Chinese Medical University, Hangzhou, China; 2 Second Affiliated Hospital, Zhejiang Chinese Medical University, Hangzhou, China

**Keywords:** 40 Hz sensory stimulation, Alzheimer’s disease, neurodegenerative disorders, cognitive impairment, non-invasive intervention

## Abstract

Alzheimer’s disease (AD) is a prevalent neurodegenerative disorder characterized by β-amyloid (Aβ) deposition, tau protein hyperphosphorylation, and synaptic dysfunction. In recent years, 40 Hz sensory stimulation—including visual, auditory, and multimodal modalities—has emerged as a novel, non-invasive intervention demonstrating potential efficacy in both animal models and preliminary clinical studies. Preclinical evidence indicates that such stimulation can markedly reduce cerebral Aβ burden (by approximately 37%–53%), inhibit tau protein phosphorylation, enhance neuronal network synchrony and synaptic plasticity, and improve learning and memory performance. Limited human trials suggest that 40 Hz sensory stimulation is safe and well tolerated in patients with mild cognitive impairment (MCI) and early-stage AD, with a slowing trend in cognitive scale score decline following intervention. This review summarizes the mechanisms of action, experimental evidence from animal models, and advances in clinical application of 40 Hz sensory stimulation in AD prevention and treatment. It further explores the potential for multimodal combination therapies integrating sensory stimulation with cognitive training, pharmacological interventions, and lifestyle modifications, and addresses challenges such as optimal timing of intervention and the influence of ambient electromagnetic fields in real-world settings. Current evidence supports 40 Hz sensory stimulation as a feasible, multi-target, and safe adjunctive intervention; however, its efficacy and applicability must be verified through multicenter, randomized controlled trials with long-term follow-up.

## 1 Introduction

Alzheimer’s disease (AD) is a prototypical age-related neurodegenerative disorder and the leading cause of dementia worldwide, accounting for more than two-thirds of all cases (GBD 2019 Dementia Forecasting Collaborators, 2022). Its pathophysiology is characterized by β-amyloid (Aβ) plaque deposition, tau protein hyperphosphorylation with neurofibrillary tangle formation, impaired synaptic plasticity, chronic neuroinflammation, and blood–brain barrier (BBB) dysfunction (Hardy and Higgins, 1992; Vanya et al., 2025; Selkoe and Hardy, 2016; Sehar et al., 2022). In the context of population aging, common comorbidities such as cardiovascular disease, type 2 diabetes mellitus, and hypercholesterolemia can alter the cerebral metabolic microenvironment and accelerate the onset and progression of AD (Livingston et al., 2020). Emerging evidence suggests that these risk factors not only intersect with AD at the pathological level, but may also undermine intrinsic neural repair capacity by influencing key signaling pathways, including Sonic hedgehog (Shh) (Parashar et al., 2024). Shh signaling remains active in the adult brain, supporting neurogenesis, synaptic network stability, and BBB integrity. Reduced Shh pathway activity has been directly linked to impaired Aβ clearance, tau-mediated neurogenic defects, and progressive cognitive decline in AD, suggesting that Shh dysregulation may represent a critical molecular basis by which aging accelerates disease progression (Parashar et al., 2024).

Driven by rapid global population aging, the number of individuals affected by AD is projected to reach 152 million by 2050, imposing an enormous socioeconomic burden on patients, families, and healthcare systems (GBD 2019 Dementia Forecasting Collaborators, 2022). Conventional pharmacotherapy offers limited benefit in slowing disease progression and is associated with adverse effects; its modest impact on cognitive decline often fails to justify the risks (Cummings et al., 2021). The limitations of drug therapy have spurred the development of neuromodulation technologies, such as transcranial magnetic stimulation (TMS), transcranial direct current stimulation (tDCS), and deep brain stimulation (DBS) (Antal et al., 2017; Lozano et al., 2019; Lefaucheur et al., 2020). These modalities show promise in modulating neural activity, improving cognition, and alleviating pathological changes, but face constraints related to cost, invasiveness, and patient tolerability—prompting interest in safe, low-cost, and widely applicable alternatives.

Recently, attention has focused on 40 Hz sensory stimulation, a non-invasive rhythmic intervention delivered through visual flicker, auditory beats, or combined multisensory modalities. This approach externally entrains cortical activity at γ-frequency (~40 Hz), synchronizing neuronal networks to modulate brain function (Chan et al., 2022; He et al., 2021; Hajós et al., 2024). Preclinical and preliminary clinical studies indicate that such entrainment can interfere with AD-related neuropathology, significantly reduce Aβ burden, and improve cognitive performance (Chan et al., 2022; Iaccarino et al., 2016). Beyond mitigating Aβ and tau pathology, γ-frequency synchronization may enhance neurophysiological function through multiple mechanisms. In animal models, *γ*-wave entrainment robustly promotes synaptic plasticity and facilitates long-term potentiation (LTP) associated with learning and memory (Adaikkan et al., 2019; Yang et al., 2023); at the cellular level, 40 Hz sensory stimulation reduces oxidative stress, improves mitochondrial respiratory chain function, and downregulates pro-inflammatory cytokine release (Adaikkan et al., 2019; Shimizu et al., 2024). From a neurovascular perspective, *γ*-frequency entrainment has been reported to enhance glymphatic clearance, promote cerebral waste removal, increase cerebral blood flow, and augment vasodilation responses, thereby improving neuronal oxygenation and metabolic support (Murdock et al., 2024; Sun et al., 2024).

Collectively, 40 Hz sensory stimulation offers a multi-target, synergistic approach for AD intervention. Its non-invasive nature, operational simplicity, and favorable safety profile confer broad translational potential. Based on this background, the present review will focus on recent advances in the use of 40 Hz sensory stimulation for AD intervention, discuss its potential clinical applications, and synthesize current evidence to inform future therapeutic strategies and research directions.

## 2 Overview of 40 Hz sensory stimulation

Forty-hertz sensory stimulation refers to the application of non-invasive, frequency-specific inputs to the brain, delivered through various sensory modalities such as vision, audition, and touch. The neurophysiological basis of 40 Hz stimulation primarily involves synchronizing neuronal activity and enhancing functional network dynamics. Neural ensembles exhibit spontaneous synchronous electrical activity at distinct frequencies, typically manifested as rhythmic oscillations in electroencephalographic (EEG) signals, a phenomenon termed neural oscillations (Başar et al., 2013). Depending on frequency, neural oscillations are classified into *δ* (1–4 Hz), *θ* (4–7 Hz), *α* (7–13 Hz), *β* (14–30 Hz), and *γ* (30–100 Hz) bands (Başar et al., 2013). Among these, γ oscillations play a pivotal role in cognitive processes, being tightly associated with perception, learning, and memory (Engel et al., 2001).

In Alzheimer’s disease and other neurodegenerative disorders, the interplay between synchronized neuronal firing and cognitive performance is markedly disrupted. Abnormal *γ* oscillations can be observed even prior to amyloid plaque formation in rodent and human models (Iaccarino et al., 2016; Etter et al., 2019). EEG and magnetoencephalography (MEG) studies further demonstrate pronounced attenuation of *γ* oscillations in the AD brain (Verret et al., 2012), coupled with impaired neuronal synchronization (Byron et al., 2021; Casula et al., 2022), resulting in a global dysregulated network state. Importantly, 40 Hz sensory stimulation has the potential to restore or augment *γ* oscillatory activity within corticohippocampal networks, analogous to a conductor reuniting a disorganized orchestra into harmonious performance. This externally driven sensory entrainment compensates for deficits in endogenous γ oscillations, a paradigm commonly referred to as Gamma Entrainment Using Sensory Stimulation (GENUS) (Cardin et al., 2009; Bánki et al., 2022; Thut et al., 2011).

## 3 Preliminary exploration of mechanisms

The neuroprotective effects of 40 Hz sensory stimulation have been validated in various animal models of AD. Its core mechanisms primarily revolve around reducing the pathological burden associated with key features of AD, such as amyloid-β deposition, tau pathology, neuroinflammation, and synaptic dysfunction. Existing evidence suggests that 40 Hz sensory stimulation can modulate neuronal network dynamics, enhance microglial phagocytic activity, and promote metabolic homeostasis, thereby slowing disease progression. Overall, these findings indicate a stable and well-defined pathological basis for the application of 40 Hz sensory stimulation in delaying the clinical course of AD. The proposed mechanism of 40 Hz sensory stimulation is illustrated in Supplementary Figure S1.

### 3.1 Reduction of neurotoxic protein deposition

Forty-hertz sensory stimulation promotes Aβ clearance and reduces its production through multiple pathways. In 2016, Iaccarino et al. (2016) first demonstrated that 40 Hz light stimulation could induce *γ* oscillations by activating fast-spiking parvalbumin-positive interneurons (FS-PV^+^). One hour of FS-PV stimulation reduced Aβ1–40 levels in the visual cortex of 5xFAD mice by 53.22%, increased Aβ1–42 by 44.62%, and enlarged the diameter of activated microglia by 1.35-fold (*p* < 0.001). Further research showed that 7 days of 40 Hz flickering light reduced senile plaque burden as verified by immunohistochemistry, whereas control groups receiving 20 Hz or 80 Hz stimulation exhibited no such changes. These effects are likely mediated via γ oscillation–enhanced microglial phagocytosis, promoting Aβ clearance while avoiding inflammation-related damage. This finding was the first to confirm the Aβ clearance efficacy of single-modality 40 Hz sensory stimulation.

Building on the visual stimulation results, Singer et al. (2018) explored auditory stimulation and found that 40 Hz auditory stimulation selectively induced *γ* oscillations in the auditory cortex (AC) and hippocampal CA1 region. In 5xFAD mice, this reduced hippocampus-dependent spatial and recognition memory error rates by 41% and 37%, respectively, and significantly decreased soluble Aβ1–42 levels in the AC (−51.8%) and hippocampus (−46.9%). Moreover, combined visual–auditory stimulation, via cross-modal γ oscillatory coupling, produced stronger γ oscillations than either modality alone, leading to marked microglial clustering within a 25 μm radius of Aβ plaques in the AC (+48.9%), visual cortex (+31.6%), CA1 (+33.1%), and medial prefrontal cortex (+38.6%). This specific “plaque-encapsulation” response activated the Nrf2 pathway, promoting myelin repair and suppressing neuroinflammation. Whole-brain analysis showed a 37% reduction in neocortical plaque volume, revealing that cross-modal *γ* entrainment can systemically enhance glymphatic clearance and microglial phagocytosis.

Recently, Tinston et al. (2025) corroborated these findings, reporting that daily one-hour 40 Hz audiovisual stimulation in 5xFAD mice reduced amyloid pathology by 50%, decreased seizure severity in an amygdala kindling model, and delayed seizure onset. In 2024, Murdock et al. (2024) further elucidated the mechanisms by which sensory stimulation enhances Aβ clearance. Administering 40 Hz audiovisual stimulation to six-month-old 5xFAD mice increased meningeal lymphatic vessel diameter by 19%, enhanced cortical tracer accumulation from cerebrospinal fluid by 34%, and boosted Aβ efflux to cervical lymph nodes by 47%. The stimulation induced astrocytic aquaporin-4 (AQP4) polarization along vascular endfeet (+28% polarity index) and activated vasoactive intestinal peptide (VIP) neurons to regulate arterial pulsation (0.1 Hz band power increase), thereby driving cerebrospinal fluid–interstitial fluid exchange and enhancing interstitial fluid clearance by 22%. Single-nucleus RNA sequencing revealed gene expression changes in endothelial cells, astrocytes, and neurons. Chemogenetic inhibition of VIP neurons abolished these effects, confirming that multimodal γ stimulation promotes systemic Aβ clearance via AQP4-dependent fluid dynamics and VIP-mediated vascular regulation.

Collectively, 40 Hz sensory stimulation reduces amyloid pathology in AD animal models through multiple mechanisms, providing a stable and well-defined pathological basis for its potential to slow disease progression.

### 3.2 Enhancement of synaptic function and neural network synchronization

Restoration of synaptic integrity is one of the core mechanisms by which 40 Hz sensory stimulation improves cognitive performance. Adaikkan et al. (2019) using the P301S tauopathy mouse model, observed that 22 days of daily 40 Hz light stimulation markedly increased *γ* power in the primary visual cortex (V1) and prefrontal cortex (PFC). In unstimulated animals, significant hippocampal neuron loss was reported, whereas neuronal numbers in the 40 Hz stimulation group were comparable to wild-type controls. Moreover, these mice demonstrated superior performance in the Morris water maze test (Adaikkan et al., 2019). Random-frequency stimulation (10–80 Hz) did not yield similar synaptic protection, confirming the frequency specificity of 40 Hz stimulation. Importantly, light flicker technology is considered more feasible for clinical translation compared with optogenetics (Singer et al., 2018).

In 2023, Yang et al. (2023) comprehensively evaluated the effect of 40 Hz sensory stimulation on synaptic plasticity across multiple AD animal models. In early-stage AD transgenic rats, continuous 40 Hz stimulation for 2 h per day over 2 weeks significantly restored impaired long-term potentiation (LTP) levels (*p* > 0.9999) to wild-type levels. LTP values were negatively correlated with hippocampal caspase-1 expression, suggesting a possible link to anti-inflammatory mechanisms. In corticosterone-induced stress models and non-transgenic rats injected with brain extracts from AD patients, 40 Hz stimulation likewise reversed LTP deficits (both *p* < 0.01), whereas random-frequency stimulation (10–80 Hz) had no measurable effects, further substantiating the specificity of the 40 Hz rhythm.

In the same year, Suk et al. (2023) applied 40 Hz tactile vibration stimulation to the Tau P301S and CK-p25 mouse models for 1 h daily over 21–42 days. This intervention significantly reduced pTau (S396)-positive cell density (−85.8% in the primary somatosensory area [SSp], −72.1% in the primary motor area [MOp]), restored neuronal NeuN expression (+124.6% in SSp), and upregulated synaptic markers vGlut1 and GABBR1 (SSp + 119.0%/+107.9%, MOp +133.9%/+143.6%). DNA damage area decreased by approximately 85%. Motor function testing revealed notable improvements in coordination and balance (Rotarod +171.7%, *p* < 0.05), indicating that 40 Hz *γ* tactile stimulation can concurrently repair synaptic structures and motor-associated neural networks.

In 2024, Barzegar Behrooz et al. (2024) employed a sporadic AD rat model induced by bilateral intracerebroventricular injection of streptozotocin (STZ). Continuous 40 Hz white light stimulation for 15 min daily over 7 days was administered, and cognitive function was assessed using the Morris water maze (MWM), novel object recognition (NOR), and passive avoidance tests. Analyses of mitochondrial metabolites, channel activity, and synaptic plasticity markers demonstrated that 40 Hz light stimulation reversed STZ-induced impairments in synaptic plasticity. Following high-frequency stimulation, the treatment group restored the slope of field excitatory postsynaptic potentials (fEPSP) and population spike (PS) amplitude in the dentate gyrus (DG) to control levels (*p* < 0.01), improved LTP, and enhanced paired-pulse facilitation (PPF), confirming that this stimulation modality effectively maintains hippocampal network synchronization and information transmission efficiency.

In summary, frequency-specific rhythmic stimulation at 40 Hz markedly improves synaptic structure and function, enhances neural network synchronization, and supports the preservation of cognitive abilities. This phenomenon has been repeatedly validated across diverse animal models, underscoring its generalizability and reproducibility.

### 3.3 Attenuation of oxidative stress and modulation of neuroinflammatory responses

Dysregulated inflammatory signaling represents a major pathogenic mechanism in AD (Hampel et al., 2018). Forty-hertz sensory stimulation activates antioxidant pathways and suppresses oxidative injury, thereby maintaining redox homeostasis within the brain. In AD mouse experiments conducted by Adaikkan et al. (2019), daily 1 h sessions of 40 Hz light stimulation for 1 week restored microglial morphology in the visual cortex and hippocampus to a state resembling that of healthy controls. Under pathological conditions in AD, microglia often remain chronically hyperactivated in a proinflammatory state, releasing cytokines such as tumor necrosis factor-*α* (TNF-α) and interleukin-1β (IL-1β), both of which are neurotoxic (Hanisch and Kettenmann, 2007). Forty-hertz stimulation inhibited excessive microglial activation and markedly reduced the levels of CD40 and complement component C1q, thereby alleviating neuroinflammation (Adaikkan et al., 2019).

In 2023, Prichard et al. (2023) further elucidated the immune-modulatory mechanism. Forty-hertz visual flicker activated NF-κB signaling in healthy neurons, inducing the secretion of immune mediators such as macrophage colony-stimulating factor (M-CSF) and interleukin-10 (IL-10). These signals promoted a phenotypic shift of microglia from a “surveillance state” to a “phagocytic clearance state” and suppressed excessive inflammatory responses. In contrast, 20 Hz stimulation maintained microglia in a patrolling morphology, highlighting the frequency-specific immune regulation of 40 Hz γ oscillations. This provides a molecular basis for the clearance of pathological substrates and the maintenance of neural homeostasis under disease conditions.

In 2024, Rodrigues-Amorim et al. (2024) employed a cuprizone-induced demyelination model in male mice. Following 3 weeks of multi-sensory 40 Hz stimulation, both the area (*p* = 0.0074) and thickness (*p* = 0.0065) of myelin in the corpus callosum were significantly preserved, while the fluorescence intensity of the myelin marker Fluoromyelin was markedly enhanced (*p* = 0.0020). Importantly, the number of activated microglia (Iba1+) and astrocytes (GFAP+) in the corpus callosum region was significantly reduced (*p* < 0.05). Additionally, key proinflammatory molecules, including complement C1q (*p* = 0.0022) and the danger-associated molecular pattern HMGB1 (*p* < 0.0001), were significantly lowered following 40 Hz intervention.

Overall, forty-hertz sensory stimulation fine-tunes microglial activation, simultaneously promoting the clearance of pathological factors while suppressing excessive inflammation, thereby mitigating oxidative damage. This dual effect forms an essential component of its neuroprotective mechanism.

### 3.4 Enhancement of metabolism and regulation of cerebrovascular function

Metabolic decline and reduced cerebral blood volume are recognized as key pathogenic factors in a range of neurodegenerative disorders, including AD. In 2019, Martorell et al. (2019) reported that in multiple AD transgenic mouse models, daily 1-h sessions of 40 Hz auditory stimulation for seven consecutive days significantly reduced soluble Aβ42 levels in the auditory cortex (−51.84 ± 4.98%) and hippocampus (−46.89 ± 3.89%; both *p* < 0.0001), as well as decreased amyloid plaque number and area (auditory cortex plaques: −45.73%, area: −54.37%; both *p* < 0.001). The intervention also induced pronounced vasodilation in the CA1 region of the hippocampus (+104.70 ± 10.96%; *p* < 0.0001) and enhanced co-localization of Aβ with vascular endothelial low-density lipoprotein receptor-related protein 1 (LRP1) in both the auditory cortex (+53.87 ± 2.70%) and CA1 (+57.78 ± 1.73%; *p* < 0.05). These findings suggest that 40 Hz stimulation not only activates microglial phagocytosis of Aβ but also facilitates vascular-mediated Aβ clearance.

In 2024, Sun et al. (2024) further demonstrated that this effect is closely associated with coupling between the cerebral lymphatic and vascular systems. In the 5XFAD mouse model, 1-h multi-sensory (light + sound) 40 Hz stimulation significantly increased cerebrospinal fluid (CSF) influx into the frontal cortex (*p* < 0.001) and enhanced interstitial fluid clearance rate (*p* < 0.01). These changes were accompanied by increased polarization index of aquaporin-4 (AQP4) in astrocytic endfeet (*p* < 0.01), augmented pulsatility of cortical arterioles (*p* < 0.01), and enlarged diameter and volume of meningeal lymphatic vessels (*p* < 0.05 and *p* < 0.01, respectively). The authors confirmed that 40 Hz visual flicker, applied to awake mice independently of sleep or anesthesia, can robustly enhance both influx and efflux through the brain’s lymphatic system.

Similarly, another study (Murdock et al., 2024) found that short-term (30 min) 40 Hz light stimulation increased cortical blood flow in multiple brain regions (*p* < 0.05), and via equilibrative nucleoside transporter 2 (ENT2)-mediated adenosine efflux, activated adenosine A₂A receptors. This downstream pathway regulated AQP4 polarization and promoted vascular pulsatility.

Collectively, 40 Hz sensory stimulation significantly augments brain metabolism and cerebral blood volume through integrated neuro-glia-vascular network dynamics. The core mechanisms involve ENT2-adenosine-A₂A receptor signaling-driven AQP4 membrane polarization, vasoactive intestinal peptide (VIP)-mediated arterial pulsation enhancement, and coordinated clearance via brain lymphatic and vascular pathways. These processes provide a potential molecular strategy to alleviate cerebrovascular and metabolic impairments in neurodegenerative diseases.

## 4 Clinical research progress of 40 Hz sensory stimulation in Alzheimer’s disease

Building upon the promising results from animal experiments, multiple clinical studies have confirmed the safety and targeted efficacy of 40 Hz sensory stimulation in populations related to AD, encompassing the full clinical spectrum from mild cognitive impairment (MCI) to mild and moderate AD. Overall, current clinical findings indicate that 40 Hz stimulation primarily benefits patients by maintaining cognitive performance and daily functional capacity, accompanied by partial improvements in pathological biomarkers. However, variability in both response magnitude and direction has been observed across different study populations, underscoring the need for more rigorous, standardized, and large-scale trials to validate and optimize therapeutic protocols. Clinical baseline and outcome data are summarized in Supplementary Table S1.

### 4.1 Safety and feasibility

Multiple clinical trials have demonstrated the high safety profile and practical feasibility of 40 Hz sensory stimulation. Chan et al. (2022) conducted a phase 2A pilot study using a single-blind, randomized controlled design. Fifteen patients with early-stage AD were enrolled and randomly assigned to a 40 Hz combined light–sound stimulation group (*n* = 8) or a sham control group (*n* = 7). Intervention consisted of 1 hour of daily home-based stimulation using synchronized devices equipped with LED panels and speakers, monitored via a camera system, over a period of 3 months. No serious adverse events were reported, with only mild drowsiness in some participants, confirming the safety and feasibility of home-based administration.

In a feasibility study of the “AlzLife” 40 Hz sensory therapy, McNett et al. (2023) recruited 27 participants with mild cognitive impairment (MCI), early-stage AD, or subjective cognitive decline. Participants received daily one-hour combined light–sound stimulation delivered through a tablet-based application for 6 months. Cortical rhythm entrainment was monitored using EEG, while cognitive changes were assessed with the Montreal Cognitive Assessment (MoCA) and Brief Orthopaedic Cognitive Assessment (BOCA) scales. Among 11 participants who completed the study, no side effects were reported; device satisfaction reached 82%, and mean adherence was 95.5%.

Agger et al. (2023) randomized 62 patients with mild to moderate AD into either an active intervention group or a placebo group. The active group received daily 40 Hz light stimulation delivered via invisible spectral flicker (ISF) technology for 6 months. Multidimensional evaluation revealed no significant difference in adverse event rates compared with placebo, and no serious stimulation-related reactions occurred.

More recently, Sato et al. (2025) conducted a prospective study in Japan evaluating the tolerability of 40 Hz amplitude-modulated auditory stimulation in healthy older adults. Using a fixed daily exposure protocol and combining subjective questionnaires with physiological monitoring, the study found high acceptability and no evident discomfort associated with tolerance thresholds.

### 4.2 Cognitive and daily function improvement

In a two-year open-label extension study, Chan et al. (2025) enrolled five patients with mild AD, including three late-onset female patients (72–87 years old, APOE E3/E3) and two early-onset male patients (54–61 years old, one APOE E3/E4). Participants received daily home-based one-hour 40 Hz audiovisual stimulation using GENUS devices. Cognitive function was assessed using five scales—MMSE, Clinical Dementia Rating (CDR), and the FAS verbal fluency test—along with EEG monitoring. Results showed that late-onset patients exhibited an annual decline in MMSE scores that was 0.8–1.2 points slower than matched controls, and achieved statistically significant improvement in CDR scores (*p* = 0.02). No clear cognitive benefits were observed in early-onset patients.

Lahijanian et al. (2024) investigated auditory gamma stimulation in dementia patients using EEG and functional neuroimaging, finding that 40 Hz auditory stimulation enhanced connectivity within the default mode network, particularly strengthening rhythmic synchrony between frontal and parietal cortices. These functional network improvements were positively correlated with better performance in memory tasks.

In a randomized controlled trial, Cimenser et al. (2021) enrolled AD patients and applied daily one-hour 40 Hz gamma sensory stimulation for 6 months. Daily living ability was evaluated with the Alzheimer’s Disease Cooperative Study–Activities of Daily Living (ADCS-ADL) scale, and sleep patterns were monitored via wrist actigraphy. While ADCS-ADL scores remained stable in the treatment group, the control group showed a mean decline of 3 points (*p* < 0.001). Basic functional domains such as dressing, eating, and communication were preserved in the treatment cohort. Moreover, participants receiving 40 Hz stimulation experienced reduced sleep fragmentation and longer deep sleep duration.

However, limitations were equally evident: several studies reported that 40 Hz audiovisual stimulation did not significantly improve visual thresholds or visuospatial memory, with cognitive gains mainly concentrated in attention and verbal fluency domains (Hsiung and Hsieh, 2024). In a human case study, Jones et al. (2019) applied 40 Hz light stimulation to individuals with suspected early-stage AD. EEG monitoring demonstrated that the intervention induced hippocampal 40 Hz neural entrainment, yet any cognitive improvement would likely require long-term observation.

### 4.3 Preservation of neural function and reduction of pathological biomarkers

In 2020, Suk et al. (2020) administered 40 Hz audiovisual stimulation to 16 patients with early-stage AD. Magnetic resonance imaging (MRI) revealed that the intervention group exhibited a smaller reduction in hippocampal volume compared with controls, along with enhanced inter-regional connectivity between the default mode network and the medial visual network.

In 2024, Da et al. (2024) randomly assigned 50 AD patients in a 2:1 ratio to an active intervention group (*n* = 33) or a sham stimulation group (*n* = 17). Participants received daily one-hour sessions of 40 Hz gamma sensory stimulation for 6 months. Automated image analysis of T1-weighted MRI was performed to segment the corpus callosum and calculate its area. Bayesian linear mixed-effects model analysis demonstrated that the corpus callosum area in the stimulation group increased by 0.20 ± 0.70%, whereas the sham group showed a reduction of 2.08 ± 0.87% (*p* < 0.02), suggesting structural support for interhemispheric information transfer.

In a recent study, Chan et al. (2025) enrolled five patients with mild AD and used S-PLEX immunoassay technology to measure plasma phosphorylated tau 217 (pTau217) levels. Two late-onset female patients showed substantial reductions in pTau217: a 47% decrease (from 27.6 pg./mL to 14.5 pg./mL) and a 19.4% decrease (from 9.78 pg./mL to 7.88 pg./mL), respectively. After adjustment for total plasma protein, the reductions remained significant at 54.9% and 19.2%. These findings provide the first evidence that non-pharmacological interventions can improve AD-related pathological biomarkers.

## 5 Challenges in clinical translation

In animal models, 40 Hz sensory stimulation has demonstrated marked efficacy in clearing pathological deposits and producing robust improvements in cognitive performance (Iaccarino et al., 2016; Başar et al., 2013; Suk et al., 2023). However, upon clinical translation, both the magnitude of therapeutic benefit and its generalizability appear reduced. The principal obstacles can be categorized into three interrelated dimensions:

### 5.1 Specific neurophysiological differences

Intrinsic interspecies differences in neuroanatomy and neurophysiology represent a fundamental cause of variability in stimulation efficacy. In transgenic mouse models of AD, 40 Hz light stimulation has been shown to effectively induce neural synchronization within the primary visual cortex (V1) and subsequently modulate global brain networks (Iaccarino et al., 2016; Singer et al., 2018). However, the externally induced gamma synchronization observed in human AD brains is markedly attenuated. Suk et al. (2020) using EEG monitoring, demonstrated that in elderly AD patients, synaptic aging and neuroinflammation reduce the activation efficiency of key neuronal pathways to only 50%–60% of that seen in young healthy adults, making it difficult to achieve equally robust oscillatory entrainment.

Such disparities may stem from the larger volume and structural complexity of the human brain, as well as age-related synaptic degeneration and inflammation, which diminish the activation efficiency of specific neuronal circuits in elderly AD patients. Consequently, strong and widespread neural synchronization, as observed in young healthy murine models, becomes challenging to replicate in human subjects.

Moreover, significant species differences also exist in the glymphatic system—a critical pathway for metabolic waste clearance. Martorell et al. (2019) demonstrated in mouse models that 40 Hz multimodal stimulation enhances cerebrospinal fluid (CSF) flow and accelerates Aβ clearance by promoting polarization of aquaporin-4 (AQP4) on astrocytic endfeet. In contrast, human studies have reported markedly lower levels of improvement. This may be partially attributable to differences in stimulation parameters, such as intensity and duration, but more likely reflects age-associated vascular stiffening and blood–brain barrier dysfunction in AD patients. These structural changes restrict effective CSF circulation, thereby reducing the clearance efficiency of the glymphatic system in the human brain.

### 5.2 Mismatch between intervention timing and disease stage

The timing of intervention is a critical determinant of the therapeutic efficacy of 40 Hz sensory stimulation. In animal experiments, stimulation is typically initiated in the early pathological stage or even before disease onset, when the glymphatic system, neurovascular unit integrity, and synaptic plasticity remain largely intact. Under such conditions, short-term outcomes tend to be more pronounced. For example, in murine models where the transition from healthy status to Alzheimer’s pathology takes approximately 3 months, Iaccarino et al. (2016) applied 40 Hz sensory stimulation to 3-month-old transgenic mice presenting only Aβ deposition without tau propagation. A two-week intervention led to significant pathological improvement. Similarly, Cimenser et al. (2021) reported cognitive improvement in short-term murine interventions that was approximately threefold greater than that observed in human studies.

In contrast, human clinical trials typically enroll symptomatic patients whose brains carry a heavy pathological burden, with irreversible neuronal and synaptic damage and diminished compensatory capacity. For instance, in the mild AD cohort studied by Chan et al. (2025), substantial tau tangles were already present at baseline; after 2 years of 40 Hz intervention, disease progression could be delayed but neuronal injury could not be reversed. Current evidence suggests that in early-stage AD animal models (characterized by low pathological burden and intact brain networks), 40 Hz sensory stimulation can effectively reduce Aβ deposition, restore synaptic function, and maintain cognitive performance (Singer et al., 2018; Garcia-Argibay et al., 2019). Conversely, in late-stage models or human patients, extensive neuronal loss, synaptic disconnection, and network disintegration markedly attenuate therapeutic efficacy (Soula et al., 2023).

Moreover, clinical patients often exhibit additional pathological conditions—including vascular alterations, sleep disturbances, and depression—that cannot be fully reproduced in animal models, further widening the translational gap between preclinical and human outcomes. Therefore, future study designs should prioritize early-stage screening and intervention, initiating therapy when the pathological burden is relatively mild. Large-scale, longitudinal clinical trials extending over multiple years will be essential to validate the long-term benefits of 40 Hz sensory stimulation in human AD populations.

### 5.3 Pathological heterogeneity in Alzheimer’s disease

Animal models of AD are typically generated through genetic engineering to induce excessive production and accumulation of Aβ, resulting in a singular pathological profile, rapid progression, and predictable disease course. For example, transgenic AD mouse strains such as APP/PS1 exhibit pronounced Aβ overproduction and deposition in the brain, leading to homogenous experimental cohorts in which the effects of interventions are more readily detectable.

In contrast, human AD pathology—particularly in patients with mild AD—is highly heterogeneous. Many individuals clinically diagnosed with “mild AD” may present cognitive impairment driven not only by Aβ pathology but also by mixed pathological processes, including tau aggregation, *α*-synuclein inclusions (Lewy bodies), and cerebrovascular lesions. Forty-hertz sensory stimulation may primarily target Aβ-related pathways, exerting limited influence on other pathological substrates.

Genetic background constitutes another important source of heterogeneity. For instance, in a clinical study by Chan et al. (2025), patients with APOE E3/E3 and APOE E3/E4 genotypes were enrolled. Post-intervention analysis revealed that only carriers of the E3/E3 genotype exhibited a significant improvement in face–name associative recall accuracy (mean increase: 3.75 points; *p* = 0.004), whereas E3/E4 carriers showed no significant benefit. Most transgenic mouse models used in preclinical research possess uniform genetic backgrounds, rendering them unable to replicate the complexity of human genetic variability. This gap may contribute to discrepancies between therapeutic efficacy observed in animal models and outcomes in human clinical trials.

## 6 Discussion

### 6.1 Differences in efficacy between early- and late-stage AD models

Existing evidence indicates that the timing of intervention critically influences the therapeutic efficacy of 40 Hz sensory stimulation. In early-stage AD animal models—characterized by low pathological burden and relatively intact neuronal network architecture—stimulation can markedly reduce Aβ deposition, restore synaptic function, and preserve cognitive performance (Garcia-Argibay et al., 2019). In contrast, in late-stage models or human patients, widespread neuronal loss, synaptic disconnection, and disintegration of brain network integrity substantially attenuate the benefits of the intervention (Soula et al., 2023).

Furthermore, clinical patients often present with heterogeneous neuropathologies and comorbid conditions—such as cerebrovascular changes, sleep disturbances, and depression—that are rarely replicated in animal models. This mismatch between preclinical models and human disease complexity may partially account for the limited translation of efficacy from bench to bedside.

Consequently, future clinical trial designs should emphasize early detection and intervention, aiming to initiate treatment during phases of minimal pathological progression. Such an approach may optimize therapeutic outcomes and maximize the potential impact of 40 Hz sensory stimulation in AD management.

### 6.2 Personalized stimulation strategies

Current literature lacks a standardized definition of specific parameters, frequencies, and intervention durations for 40 Hz sensory stimulation. This gap has prompted an array of personalized approaches aimed at optimizing intervention modalities, enhancing user experience, and enabling adaptive regulation, thereby broadening its clinical applicability.

On one hand, novel stimulation modalities and parameter optimization have yielded promising results. For example, a double-blind randomized controlled trial demonstrated that daily 30-min sessions of 40 Hz transcranial vibroacoustic stimulation (tVAS) for eight consecutive weeks significantly improved cognitive function and alleviated depressive symptoms in older adults. These improvements were accompanied by increased gamma-band power in electroencephalograms (EEGs), as well as enhanced amplitudes of N100 and P200 components—indicating boosted neuroplasticity and sensory processing efficiency (Bae et al., 2025). Future work may explore multimodal sensory combinations—such as integrating visual, auditory, tactile, and even olfactory stimulation. One study in healthy older adults employed 40 Hz auditory stimulation with amplitude modulation separating human voice and background music, achieving a high adherence rate of 96.4%, with 85.7% indicating willingness for continued use, and only mild device-related adverse events (Sato et al., 2025).

On the other hand, optimizing tolerability and personalization is critical. Traditional long-term visual stimulation in 40 Hz audiovisual interventions poses risks for individuals with photosensitive epilepsy, while pure auditory 40 Hz tones often produce “buzz-like” noise that can cause discomfort, limiting long-term use. To address these issues, some investigators have combined 40 Hz auditory stimulation with music to improve tolerability. For instance, Yokota et al. (2023) integrated 40 Hz tonal energy into “gamma music,” revealing significantly higher gamma-band power and phase-locking index (PLI) in drums, bass, and keyboard tracks compared with regular music, with keyboard sounds showing the strongest auditory steady-state response (ASSR) induction and improved subjective relaxation. Similarly, Tichko et al. (2022) embedded synchronous gamma-frequency visual stimulation (SynG) into natural music listening. Although single-session trials did not attain statistical significance, participants exhibited more accurate performance in visual working memory tasks. This multimodal integration leverages the inherent advantages of music while mitigating discomfort through personalized audio design, offering a more acceptable intervention format.

Adaptive closed-loop systems further demonstrate substantial potential for precision delivery. Reis et al. (2025) showed that using virtual reality (VR) headsets to monitor gamma power in real time via EEG enables dynamic adjustment of stimulation parameters. Future development may incorporate wearable devices or home-based platforms to allow intelligent parameter recommendations, remote monitoring, and online adjustments, advancing safe and scalable long-term individualized interventions.

Given the substantial interindividual variability in baseline brain network states, pathological burden, and sensory sensitivity, future clinical trials should integrate neuroimaging or EEG biomarkers to characterize personalized response profiles. This would guide tailored stimulation parameters, facilitating truly precision-based therapeutic strategies for 40 Hz sensory stimulation.

### 6.3 Electromagnetic field environment, safety, and combined use

In modern society, individuals are almost inseparable from various electronic devices—mobile phones, Wi-Fi routers, computers, household appliances—all of which generate different types of electromagnetic fields (EMFs). These EMFs are omnipresent in our daily surroundings, meaning that 40 Hz sensory stimulation experiments or clinical interventions inevitably occur within a background EMF environment. In other words, it is practically impossible to eliminate EMF exposure in real-world settings. This raises an important question: could such background signals influence our cognition?

Accumulating evidence from basic and cellular studies suggests that extremely low-frequency electromagnetic fields (ELF-EMFs) and low-intensity pulsed electromagnetic fields (PEMFs) confer multi-target therapeutic benefits in Alzheimer’s disease and other neurodegenerative disorders. For instance, in the 3xTg-AD mouse model, prolonged ELF-EMF exposure not only improved spatial learning and memory but also significantly reduced multi-site hyperphosphorylation of tau protein in the hippocampus. Mechanistically, these effects were mediated by inhibiting the activities of glycogen synthase kinase-3β (GSK-3β) and cyclin-dependent kinase 5 (CDK5), and activating protein phosphatase 2A (PP2A), thereby attenuating pathological progression, decreasing oxidative stress, and enhancing synaptic protein expression to safeguard neuronal survival (Hu et al., 2016).

Further evidence from ischemic brain injury models has demonstrated that ELF-EMF can promote neurogenesis in the dentate gyrus, upregulate Notch signaling activity, and improve synaptic plasticity, ultimately restoring cognitive performance (Gao et al., 2021). *In vitro* studies have shown that low-intensity PEMF can restore catalase activity, reduce reactive oxygen species (ROS), maintain mitochondrial membrane potential under oxidative or amyloid-induced injury conditions, and enhance neurotrophic support by suppressing aberrant extracellular signal-regulated kinase 1/2 (ERK1/2) activation and upregulating the cyclic AMP/cAMP response element-binding protein/brain-derived neurotrophic factor (cAMP/CREB/BDNF) signaling pathway. These changes collectively mitigate apoptosis and inflammatory responses (Merighi et al., 2024).

Moreover, in APP/PS1 mice with varying degrees of cognitive impairment, 40 Hz pulsed magnetic field stimulation has been reported to improve cognition and mitochondrial dynamic homeostasis while augmenting autonomic nervous function, suggesting promise for individualized neurofeedback interventions (Zhang et al., 2025). Given the overlapping anti-inflammatory, antioxidant, and neuroplasticity-enhancing pathways shared by ELF-EMF/PEMF exposure and sensory stimulation, systematic evaluation of their integration in multimodal therapeutic frameworks is warranted.

### 6.4 Rationale for multimodal stimulation therapy

It is noteworthy that 40 Hz sensory stimulation is not an isolated therapeutic approach; when combined with other interventions, it may further enhance efficacy. Clinical evidence indicates that regular physical exercise can improve cognitive function in patients with Alzheimer’s disease by promoting hippocampal neurogenesis and synaptic plasticity (Nichol et al., 2008; Du et al., 2018). In murine studies, researchers have demonstrated that combining 40 Hz light stimulation with voluntary wheel running reduced hippocampal Aβ levels in 3xTg-AD mice to nearly one-quarter of control values, restored the phosphorylated tau/tau ratio to normal, and achieved efficacy superior to either intervention alone (Park et al., 2020).

In addition, modulation of disrupted circadian rhythms has been shown to synergize with 40 Hz light stimulation. One animal study revealed that 30 days of morning 40 Hz light exposure increased the rhythm amplitude index (RA) in AD model mice from 0.45 to 0.82, while concurrently reducing brain Aβ42 and phosphorylated tau protein levels by 41% and 34%, respectively. This finding suggests a novel strategy for optimizing Aβ clearance through rhythm synchronization in AD patients with concomitant sleep disturbances (Yao et al., 2020).

Strong synergistic effects of multifactorial combined stimuli have also been documented. In APP/PS1 mice, reduced adult neurogenesis is closely related to behavioral abnormalities in AD. A 4-week regimen combining treadmill exercise, 40 Hz auditory–visual stimulation, and olfactory stimulation significantly increased hippocampal newborn cells, immature neurons, and astrocytes, decreased Aβ burden, improved cognition, alleviated depressive behavior, and maintained the consolidation effect on immature neurons for at least 2 weeks after treatment cessation (Xiao et al., 2025).

These preclinical findings indicate that multimodal combined interventions can exert synergistic effects across multiple pathological processes, including neurogenesis, synaptic plasticity, and pathological protein clearance. Clinically, coupling 40 Hz rhythmic stimulation with cognitive training not only robustly amplifies cortical gamma-frequency entrainment effects but also induces significant synchronization responses in deep brain nuclei such as the hippocampus and insula (Khachatryan et al., 2022). Beyond exercise, rhythm modulation, and cognitive training, there is theoretical potential for combining pharmacotherapy with 40 Hz rhythmic stimulation. The mechanisms of 40 Hz stimulation—enhancing neural network synchronization, promoting Aβ clearance, and inhibiting pathological tau phosphorylation—complement the action targets of certain anti-AD drugs, including cholinesterase inhibitors (e.g., donepezil) and recently approved anti-Aβ monoclonal antibodies (Dichgans et al., 2008; Folch et al., 2018; van Dyck et al., 2023).

Although direct evidence from combined human or animal studies remains limited, mechanistic insights suggest that superimposing rhythmic stimulation during stable drug therapy phases could further strengthen synaptic plasticity and metabolic clearance pathways in the brain, thereby improving overall treatment efficacy. This multi-modal, synergistic therapeutic framework offers broad potential for tailoring intervention combinations to diverse patient populations and may increase sustainability and real-world applicability in clinical translation.

## 7 Future perspectives

Based on current findings from animal experiments and preliminary clinical studies, 40 Hz sensory stimulation demonstrates a promising multi-target potential in the intervention of AD. Future research can advance in the following directions:

First, optimization of clinical application parameters and delivery modes is crucial. Although this technique has achieved significant results in animal models, the optimal stimulation conditions for human patients remain to be established. Large-scale, multicenter randomized controlled trials (RCTs) should systematically evaluate the efficacy and safety of different sensory modalities—visual, auditory, and tactile—and combinations of stimulation parameters. Furthermore, incorporating disease stage and individual patient characteristics into the design of personalized intervention strategies may improve specificity and long-term sustainability.

Second, systematic exploration of combination therapy is warranted. Potential synergistic applications include pairing 40 Hz sensory stimulation with existing pharmacological agents such as cholinesterase inhibitors and anti-Aβ monoclonal antibodies, as well as integrating it with non-pharmacological strategies like cognitive training and physical rehabilitation. Such multimodal approaches could enhance efficacy while minimizing adverse effects.

Third, investigation of therapeutic effects across different disease stages is recommended to identify optimal application windows for mild, moderate, and severe AD patients.

Fourth, technological innovation will be essential to promote clinical translation. Development of portable, intelligent stimulation devices, coupled with virtual reality and artificial intelligence tools, could enable real-time feedback and closed-loop parameter adjustments, thereby improving patient compliance and overall user experience.

Finally, further research should deepen understanding of AD pathophysiology and the specific targets of 40 Hz sensory stimulation. Studies at the molecular, cellular, and neural network levels will help elucidate intervention pathways, providing a robust theoretical basis for optimizing therapeutic strategies and device design.

## 8 Conclusion

Forty-hertz sensory stimulation demonstrates multi-level therapeutic potential in the prevention and treatment of AD. Evidence from animal models and early-stage clinical trials indicates that this intervention can promote Aβ clearance, suppress excessive tau phosphorylation, improve neural network synchrony, and reduce neuroinflammatory responses. Its safety profile has been preliminarily confirmed within commonly applied stimulation parameters. When integrated into multimodal therapeutic frameworks—such as pharmacological treatment, cognitive training, and physical rehabilitation—it holds promise for enhanced efficacy.

Current data suggest that 40 Hz stimulation may yield greater benefits when applied during the early stages of disease progression. Nevertheless, large-scale, long-term clinical studies are still required to determine the optimal stimulation patterns, application windows, and population-specific suitability. With ongoing technological refinement and diversification of application contexts, 40 Hz sensory stimulation has the potential to become an essential component of comprehensive AD management strategies.
