# Supplementary material for: Research progress on 40 Hz sensory stimulation for the treatment of Alzheimer’s disease
Source: Front Aging Neurosci. 2026 Jan 16;17:1710041. doi: 10.3389/fnagi.2025.1710041 (PMC12855508; doi:10.3389/fnagi.2025.1710041)
Supplement: Supplementary file 1 [file Data_Sheet_1.docx]

| **First Author (Year)** | **Status** | **Sample Size (n)** | **Intervention Type** | **Frequency & Parameters** | **Intervention Duration** | **Primary Outcome Measures** | **outcomes** |
| --- | --- | --- | --- | --- | --- | --- | --- |
| Jones M (2019) | Completed | 8 | Light stimulation | 40 Hz, single to short-term | Case study | Cognitive observation, EEG | Induced gamma waves; increased synchrony |
| Suk H‑J (2020) | Completed | 12 | Audiovisual stimulation | 40 Hz | Single/short-term | EEG, safety | Safely induced gamma synchrony in both healthy individuals and AD patients |
| Cimenser A (2021) | Completed | 74 | Combined light + sound stimulation | 40 Hz, 1 h/day | 12 weeks | Functional ability, sleep architecture | Maintained functional ability; reduced sleep fragmentation |
| Chan D (2022) | Completed | 15 | Combined light + sound stimulation | 40 Hz, 1 h/day | 6 months | ADAS-Cog, EEG, MRI volumetry | Good feasibility; partial cognitive improvement; enhanced 40 Hz neural oscillations |
| Tichko P (2022) | Completed | 15 | Music + gamma stimulation | 40 Hz embedded in music | 4 weeks | Subjective cognition, task performance | Partial task performance improvement in MCI participants |
| Yokota Y (2023) | Completed | 20 | 40 Hz music stimulation | 40 Hz tone embedded in music | Single/short-term | Auditory steady-state response | Stable induction of gamma steady-state response |
| McNett S D (2023) | Completed | 40 | Combined light + sound (AlzLife system) | 40 Hz, 30–60 min/day | 8 weeks | Memory tests, sleep quality | Trend toward improved memory and sleep quality |
| Agger M P (2023) | Recruiting | 80 | Light stimulation system | 40 Hz, adjustable duty cycle | 3 months | Cognitive testing, neuroimaging | Results pending |
| Hsiung P C (2024) | Completed | 16 | Light + sound stimulation | 40 Hz, visual threshold tasks | Single session | Visual threshold, spatial memory | No significant enhancement observed |
| Da X (2024) | Completed | 20 | Light stimulation system | 40 Hz, continuous | 6 months | MRI structural measures, cognitive scales | Corpus callosum structural preservation; maintained cognition |
| Lahijanian M (2024) | Completed | 25 | Auditory stimulation | 40 Hz, 20 min/session, 1–2×/day | 4 weeks | DMN connectivity, MoCA | Enhanced DMN connectivity; cognitive improvement |
| Bae J H (2025) | Completed | 60 | Transcranial vibroacoustic stimulation | 40 Hz, 20 min/session, 3×/week | 8 weeks | Neuropsychological testing, memory tasks | Significant cognitive and psychological improvements |
| Reis C (2025) | Completed | 20 | VR + combined light + sound stimulation (closed-loop) | 40 Hz | 2 weeks | Feasibility, acceptability, cognitive scales | Good feasibility and acceptability; partial cognitive improvements |
| Sato S (2025) | Completed | 30 | Auditory stimulation (amplitude-modulated) | 40 Hz, continuous playback | Single session/short-term | Safety, acceptability | Safe and well tolerated in healthy elderly participants |
| Chan D (2025) | Completed | 12 | Combined light + sound stimulation (open-label extension) | 40 Hz, 1 h/day | 12 months | Cognitive scales, MRI | Prolonged intervention maintained partial functional indicators |
