# Supplementary figures and images for: Research progress on 40 Hz sensory stimulation for the treatment of Alzheimer’s disease
Source: Front Aging Neurosci. 2026 Jan 16;17:1710041. doi: 10.3389/fnagi.2025.1710041 (PMC12855508; doi:10.3389/fnagi.2025.1710041)

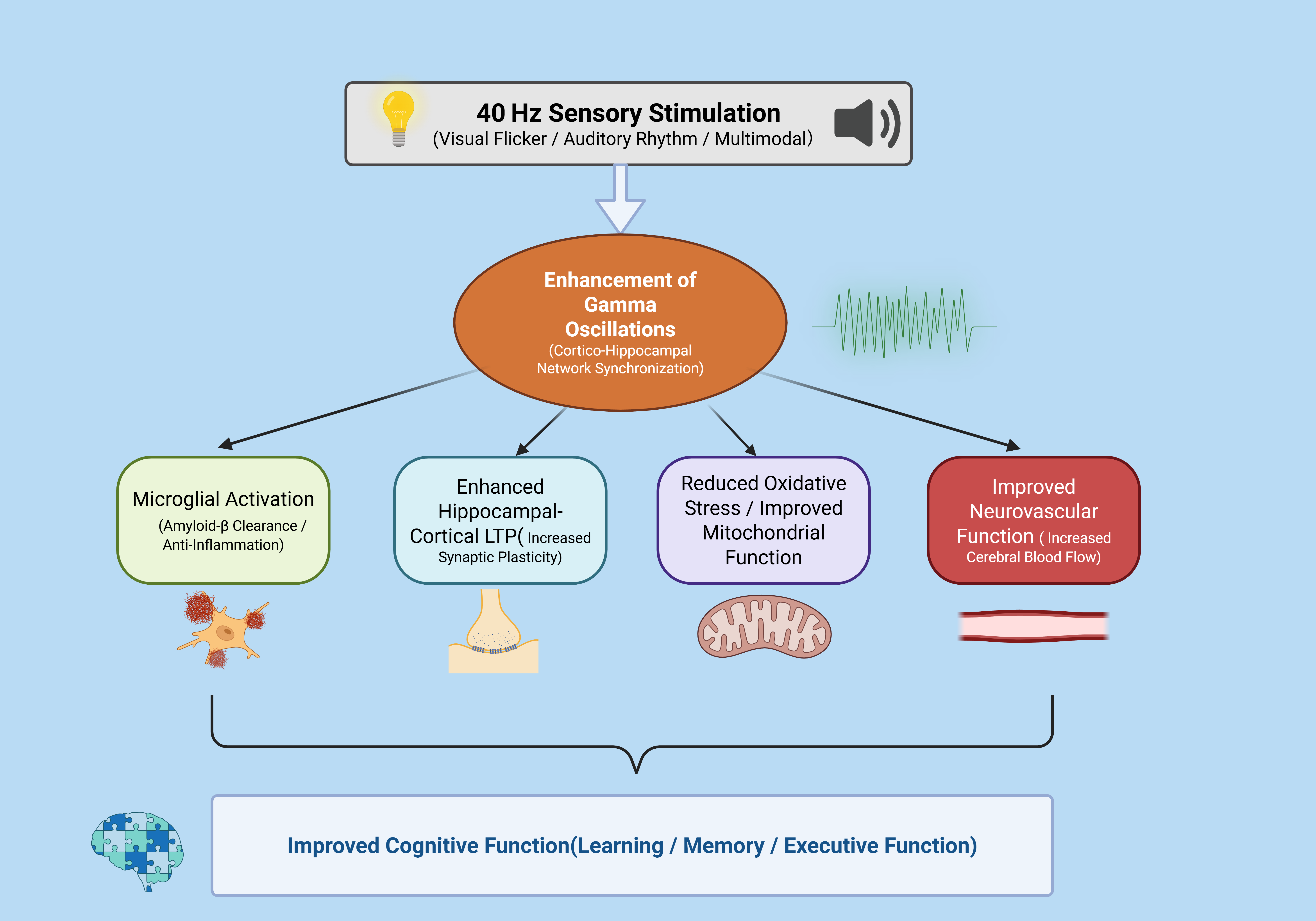

Supplement: Supplementary file 2 [file Image_1.png]
